# A Cell Differentiation Trajectory-Related Signature for Predicting the Prognosis of Lung Adenocarcinoma

**DOI:** 10.1155/2022/3483498

**Published:** 2022-08-16

**Authors:** Fan Yang, Yan Zhao, Xiaohan Huang, Jin Zhang, Ting Zhang

**Affiliations:** ^1^Department of Thoracic Surgery, Chongqing General Hospital, Chongqing 401120, China; ^2^Department of Respiratory Medicine, Second Affiliated Hospital of Chongqing Medical University, Chongqing 400000, China; ^3^Department of Respiratory and Critical Care Medicine, The First Affiliated Hospital of Army Military Medical University, Chongqing 400038, China

## Abstract

**Objective:**

To screen the cell differentiation trajectory-related genes and build a cell differentiation trajectory-related signature for predicting the prognosis of lung adenocarcinoma (LUAD).

**Methods:**

LUAD single cell mRNA expression profile, TCGA-LUAD transcriptome data were obtained from GEO and TCGA databases. Single-cell RNA-seq data were used for cell clustering and pseudotime analysis after dimensionality reduction analysis, and the cell differentiation trajectory-related genes were acquired after differential expression analysis conducted between the main branches. Then, the consensus clustering analysis was carried out on TCGA-LUAD samples, and the GSEA analysis was performed, then the differences on the expression levels of immune checkpoint genes and immunotherapy response were compared among clusters. The prognostic model was constructed, and the GSE42127 dataset was used to validate. A nomogram evaluation model was used to predict prognosis.

**Results:**

Two subsets with distinct differentiation states were found after cell differentiation trajectory analysis. TCGA-LUAD samples were divided into two cell differentiation trajectory-related gene-based clusters, GSEA found that cluster 1 was significantly related to 20 pathways, cluster 2 was significantly enriched in three pathways, and it was also shown that clusters could better predict immune checkpoint gene expression and immunotherapy response. A six cell differentiation-related genes-based prognostic signature was constructed, and the patients in the high-risk group had poorer prognosis than those in the low-risk group. Moreover, a nomogram was constructed based on the prognostic signature and clinicopathological features, and this nomogram had strong predictive performance and high accuracy.

**Conclusion:**

The cell differentiation-related signature and the prognostic nomogram could accurately predict survival.

## 1. Introduction

Lung cancer is a common cancer with the highest incidence rate and mortality rate worldwide [1]. The incidence rate and mortality rate of lung cancer in China have been increasing in recent years, and it is reported that the incidence and mortality of lung cancer in China are 17.9% and 23.8% in 2020, respectively [2]. Lung cancer can be categorized into small cell lung cancer and nonsmall cell lung cancer according to the different histopathological characteristics [3]. Lung adenocarcinoma (LUAD) is the most common type of lung cancer, which belongs to nonsmall cell lung cancer, accounting for 45.5% of lung cancer [4]. LUAD is also a heterogeneous tumor, and its biological behavior is affected by a complex intracellular gene regulatory network. Although the treatment of lung cancer has made good progress in recent years, the survival rate of LUAD is still not ideal, and the five-year survival rate of LUAD is only 20–30% [5, 6].

In the process of cell division and differentiation, owing to the reprogramming of genome and epigenome and DNA replication errors, individual cells can present different genomes, transcriptome and epigenome [7]. The development of single-cell RNA-seq offers an opportunity to comprehensively describe genetic complexity at the cellular level, containing copy number variations, gene fusions, gene expression levels, etc. [8]. Cell differentiation is closely related to tumorigenesis, with certain genes potentially functioning as differentiation regulators. The ability of cells to respond to cell-cell contact by restraining cell circulation and further circulation depends on differentiation. This ability loss is a feature hallmark in tumorigenesis which is due to a failure of differentiation [9, 10]. However, the cell differentiation-related genes and cell differentiation-related signature in LUAD have been rarely reported.

Thus, in this study, based on the LUAD single-cell mRNA expression profile and TCGA-LUAD transcriptome data, we screened the cell differentiation trajectory-related genes and built a cell differentiation trajectory-related signature for predicting the prognosis of LUAD. This study provides novel insights and therapeutic targets for LUAD.

## 2. Materials and Methods

### 2.1. Data Collection

The LUAD single-cell mRNA expression profile of the GSE149655 dataset was obtained from the GEO database, among which two LUAD samples were analyzed in this study, containing GSM4506699 and GSM4506701. Besides, the TCGA-LUAD transcriptome and clinical data were acquired from the UCSC database (http://genome.ucsc.edu/) to perform consensus clustering analysis and construction of the prognosis model, and the GSE42127 dataset including 176 LUAD samples was used as the validation set of the prognostic model.

### 2.2. Data Preprocessing and Principal Component Analysis (PCA) Dimensionality Reduction Analysis

The quality control and statistical analysis of single-cell RNA-seq data were conducted utilizing the Seurat package [11, 12] in R. Filter according to the following quality control standards: (a) genes found in <5 cells were filtered out; (b) cells with a total number of detected genes <200 were filtered out; (c) cells with mitochondrial gene expression ≥25% were filtered out. The data in the GSE149655 dataset were logarithmically normalized, and the PCA was used to determine the available dimensions and screen-related genes.

### 2.3. Cell Clustering and Pseudotime Analysis

Unsupervised cluster analysis of cells was carried out by using the function of FindNeighbors and FindClusters in the Seurat package, and the nonlinear dimensionality reduction method was used. In addition, the scCATCH [13] in R was employed to annotate the cell type in the cluster. Moreover, the pseudotime on single-cell data was performed using Monocle [14]. Then, the differential expression analysis was conducted between the main branches with the cutoff value of |log2FC| ≥ 1 and *P* < 0.01, and the cell differentiation trajectory-related genes were acquired, which were considered as the marker genes.

### 2.4. Consensus Clustering Analysis on TCGA Samples

The consensus clustering analysis on the TCGA samples (based on the marker genes in each branch) was carried out using the ConsensusClusterPlus package [15] with the parameters of clusterAlg = ‘km dist', distance = ‘euclidean', and repeat times = ‘1000'. The Chi square test was utilized to evaluate the significant differences in the distribution of clinical characteristics (tumor stage and age) of different subtypes.

### 2.5. Gene Set Enrichment Analysis (GSEA)

Total 51 hallmark gene sets were downloaded from the molecular signatures database (MSigDB), then clusterprofiler (version: 3.8.1, http://bioconductor.org/packages/release/bioc/html/clusterProfiler.html) in R was employed to determine whether any signatures were enriched in two clusters by GSEA analysis based on TCGA-LUAD expression matrix. Significantly enriched hallmarks were chosen according to a *P* value < 0.05.

### 2.6. Immune Checkpoint Genes and Immunotherapy Response across Two Clusters

The differences of the six immune checkpoint expression levels among different subtypes were evaluated. The possibility of subtypes responding to immunotherapy was predicted using the submap algorithm in TIDE [16] and GenePattern [17] websites.

### 2.7. Generation and Validation of a Prognostic Model

The single-factor Cox proportional hazards regression analysis was performed on the marker genes in TCGA-LUAD, and the genes with the cutoff value of *P* < 0.05 were screened. Then, multivariate Cox analysis and stepwise regression were carried out to identify the prognosis-related genes, and after that the prognostic model was built. To assess the prognosis value of the prognostic model, the risk score was calculated using predict function with the following formula: riskscore=*h*_0_(*t*)^*∗*^exp(*β*_1_*X*_1_+*β*_2_*X*_2_+…+*β*_*n*_*X*_*n*_) (*β*: regression coefficient, *h*_0_(t): benchmark risk function, exp: the nth power of natural number). The patients were categorized into high-risk and low-risk groups based on the median risk score value. Besides, the GSE42127 dataset was utilized to validate the prognostic model.

## 3. Results

### 3.1. Quality Control and PCA Dimensionality Reduction Analysis

**Figure 1:**
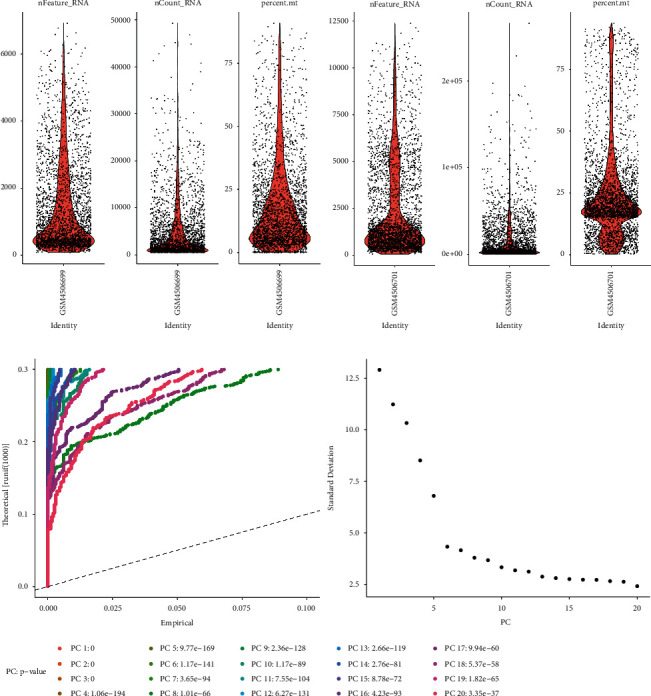
Quality control and normalization of scRNA-seq data and dimensionality reduction analysis. (a) After quality control and normalization, 2,962 cells were screened for further analysis. (b) Total 20 PCs with significant differences were identified with *P* < 0.05.

After filtering according to the quality control standards, a total of 2,962 cells were included in this study. The number of detected genes was correlated with sequencing depth, with a total of 20,240 corresponding genes (Figure 1(a)). Then, the PCA was used to determine the available dimensions and identify the related genes and a total of 20 principal components (PCs) with the threshold of *P* < 0.05 (Figure 1(b)).

### 3.2. Cell Clustering and Pseudotime Analysis

**Figure 2:**
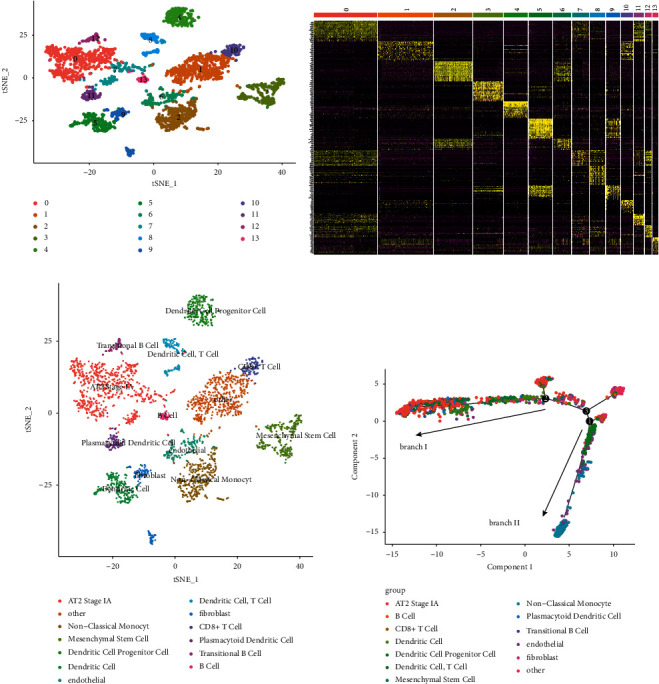
Cell clustering and pseudotime analysis. (a) Principal component analysis (PCA) based on scRNA-seq data. (b) Total 2,962 cells were aggregated into 14 clusters and the top 20 of marker genes in each cluster are displayed on the heatmap. (c) Cell-type identification of each cluster. (d) Pseudotime and trajectory analysis.

Unsupervised cluster analysis of the 2,962 cells was carried out by using the function of FindNeighbors and FindClusters in the Seurat package. Then, the FindClusters tool was used to cluster the cells (Figure 2(a)), and the heatmap of the top 20 genes in the cell cluster is shown in Figure 2(b). In addition, scCATCH in R was employed to annotate the cell type in clusters (Table 1; Figure 2(c)). Besides, two main branches (branch I and branch II) were obtained (Figure 2(d)), and the cell differentiation trajectory-related genes in each branch were acquired after differential expression analysis, which were considered as the marker genes. Then, the 38, 93 marker genes in branch I and branch II were obtained, respectively, and the total of 131 marker genes were used for subsequent analysis.

### 3.3. Two Cell Differentiation Trajectory-Related Gene-Based Clusters from TCGA Dataset

**Figure 3:**
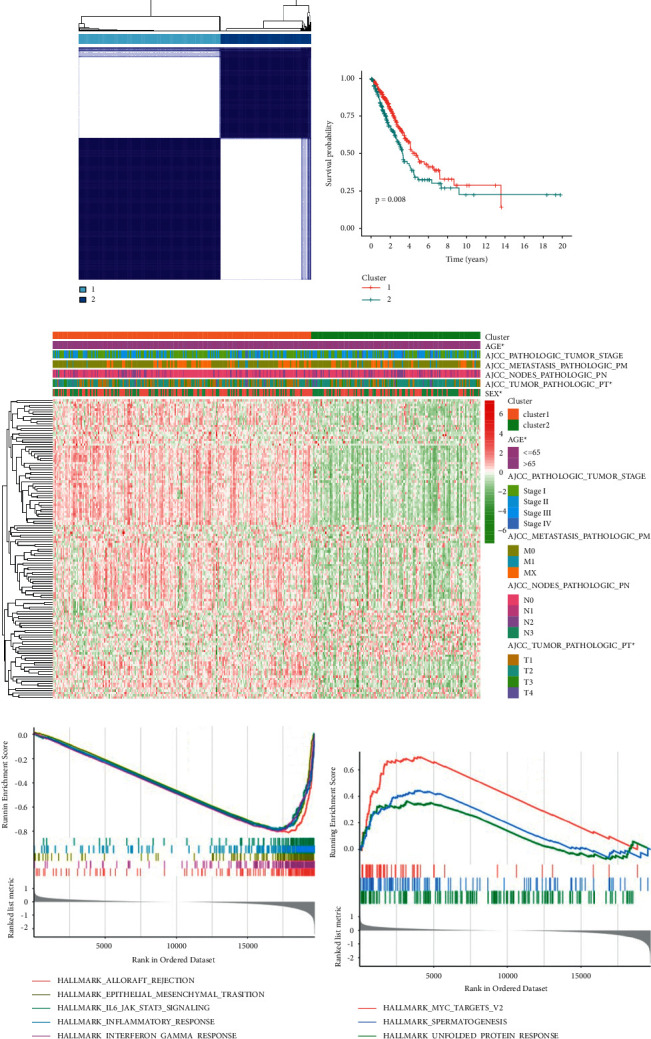
Cell differentiation trajectory-related gene-based consensus clustering analysis on TCGA samples. (a) Two molecular clusters were identified. (b) Kaplan–Meier analysis between the two clusters. (c) The heatmap of the relationships of clusters and clinic information. GSEA revealed that the pathways were involved in cluster 1 (d) and cluster 2 (e).

Based on the expression pattern of 131 marker genes, the consensus clustering analysis was carried out on TCGA samples by utilizing the ConsensusClusterPlus package, and total two clusters were obtained, including cluster 1 and cluster 2 (Figure 3(a)). The Kaplan–Meier analysis showed that the overall survival (OS) of LUAD patients in cluster 1 was higher than that in cluster 2 (Figure 3(b)). The heatmap of the relationships of clusters and clinic information is shown in Figure 3(c). GSEA found that cluster 1 was significantly related to 20 pathways, including allograft rejection, epithelial mesenchymal transition, IL6-JAK-STAT3 signaling, and so on, and the top 5 involved pathways are shown in Figure 3(d). Cluster 2 was significantly enriched in three pathways, containing MYC-targets-V2, spermatogenesis, and unfolded protein response (Figure 3(e)).

### 3.4. Expression Pattern of Immune Checkpoints and Immunotherapy Response

**Figure 4:**
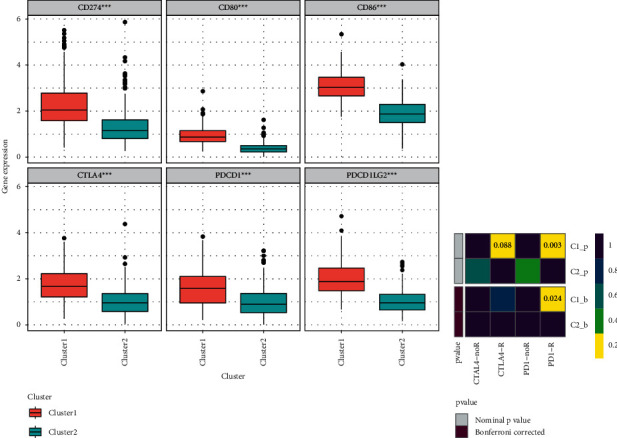
Comprehensive analysis of the expression pattern of immune checkpoints and immunotherapy response across two clusters. (a) Six immune checkpoints; (b) immunotherapy response.

The expression levels of six immune checkpoints among different subtypes were compared, and the results are shown in Figure 4(a); the expression levels of the six immune checkpoint genes significantly increased in cluster 1 when compared to that in cluster 2. In addition, the possibility of subtypes responding to PD1 and CTLA4 inhibitors was predicted using the submap algorithm in TIDE and GenePattern websites, and the result showed that LUAD patients in cluster 1 were more sensitive to anti-PD1 therapy (Figure 4(b)).

### 3.5. Construction of the Prognostic Model

**Figure 5:**
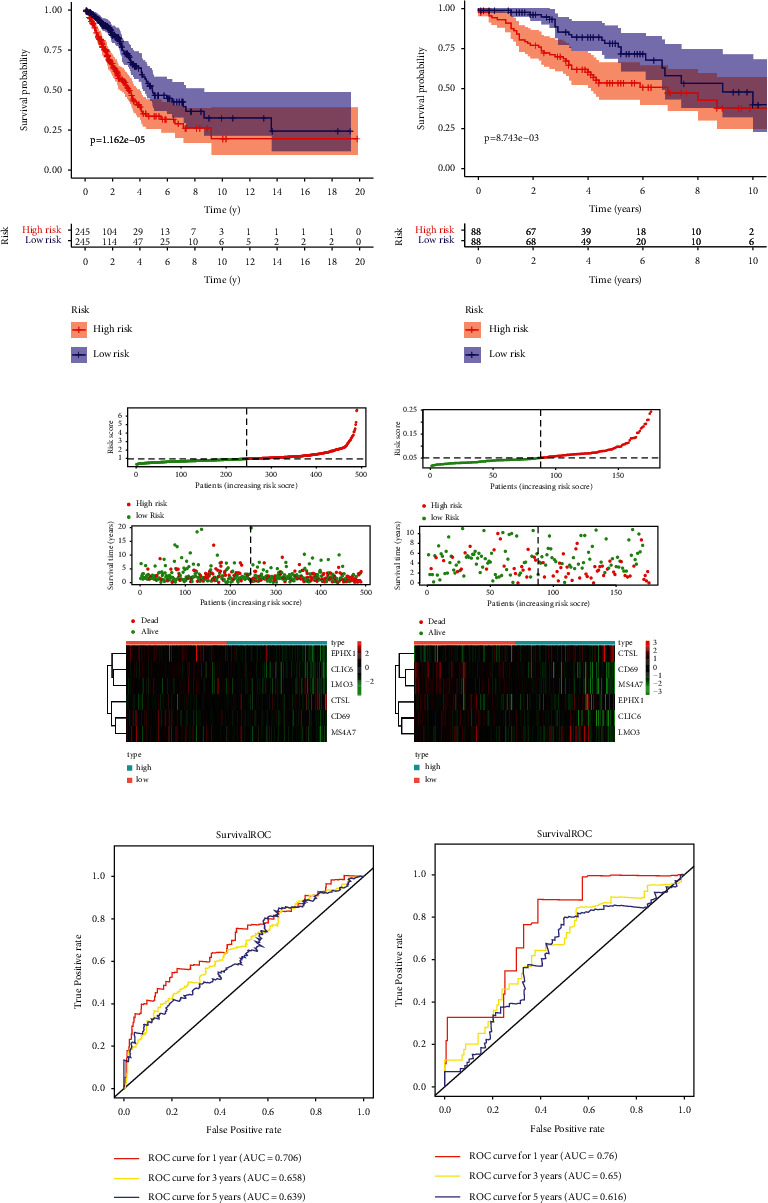
Construction and validation of the prognostic model. Kaplan–Meier analysis shown that patients in the high-risk group had poorer prognosis than those in the low-risk group based on TCGA dataset (a) and GSE42127 dataset (b). The distribution of the risk score, the survival status, and the expression of six prognosis-related genes in the TCGA (c) and GSE42127 dataset (d). Receiver operating characteristic (ROC) curves for predicting overall survival (OS) at 1-year, 3-year, and 5-year for TCGA dataset (e) and GSE42127 dataset (f).

Then, based on the expression matrix of 131 marker genes in TCGA-LUAD, six prognosis related genes were utilized to build the prognostic model, containing CD69, CLIC6, CTSL, EPHX1, LMO3, and MS4A7. Then, the risk score was calculated with the following formula: risk score = 2.54805945883909 *∗* exp [(−0.145125727) *∗* CD69 + (−0.103143891) *∗* CLIC6 + 0.287650127 *∗* CTSL + (−0.207269964) *∗* EPHX1 + 0.101089343 *∗* LMO3 + (−0.279120242) *∗* MS4A7]. The Kaplan–Meier analysis showed that patients in the high-risk group had poorer prognosis than those in the low-risk group (*P* < 0.05) (Figures 5(a) and 5(b)). The distribution of the risk score, survival status, and the expression of six prognosis-related genes in TCGA and GSE42127 datasets are displayed in Figures 5(c) and 5(d). As for OS, the AUCs at 1-year, 3-year, and 5-year were 0.706, 0.685, and 0.639 for TCGA dataset and 0.76, 0.65, and 0.616 in the GSE42127 dataset (Figures 5(e) and 5(f)).

### 3.6. Construction of a Nomogram for Predicting Patient OS

**Figure 6:**
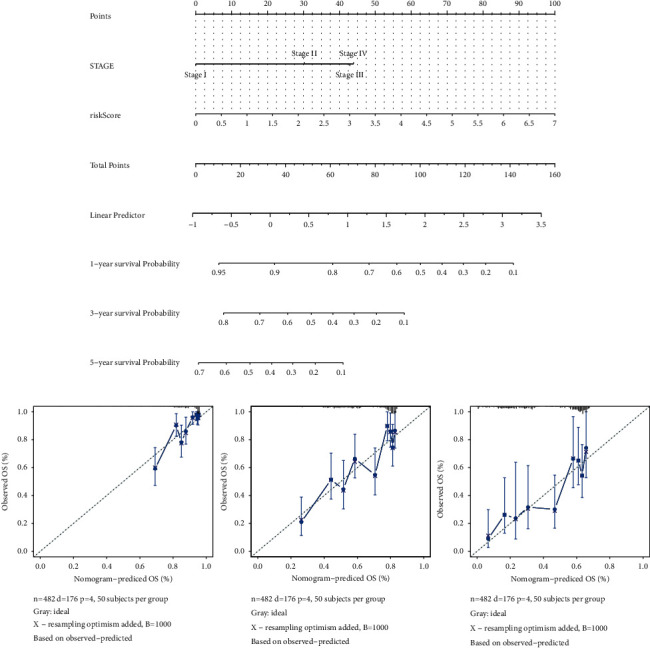
Construction and evaluation of a nomogram. (a) A nomogram for predicting 1-year, 3-year, and 5-year overall survival (OS). (b) The calibration curves for predicting 1-year, 3-year, and 5-year OS.

To explore the relation between clinicopathological features and the prognosis model, age, sex, MNT, stage, and the risk score in TCGA-LUAD samples were analyzed, and the result uncovered that stage and the risk score were independent prognostic factors in patients with LUAD (*P* < 0.01; Table 2). Besides, a nomogram was built with the stage and risk score (Figure 6(a)), and the calibration plots revealed that the nomogram might accurately estimate the mortality (Figure 6(b)).

## 4. Discussion

It has been suggested that cancer should be regarded as a disease of cell differentiation [10]. Thus, this study aimed to screen the cell differentiation trajectory-related genes and built a cell differentiation trajectory-related signature for predicting the prognosis of LUAD. A total of 131 cell differentiation trajectory-related genes were obtained, then consensus clustering analysis was conducted on TCGA samples, and total two clusters were obtained, including cluster 1 and cluster 2. The Kaplan–Meier analysis showed that the OS of LUAD patients in cluster 1 was higher than that in cluster 2. GSEA found that cluster 1 was significantly related to 20 pathways, including allograft rejection, epithelial mesenchymal transition, and IL6-JAK-STAT3 signaling, and cluster 2 was significantly enriched in three pathways, containing MYC-targets-V2, spermatogenesis, and unfolded protein response. The cell-biological program termed the epithelial mesenchymal transition plays an important role in both development and cancer progression [18]. Numerous studies have found that the IL6-JAK-STAT3 signaling pathway was activated abnormally in a variety of tumor tissues, which has an immense influence on tumor progression [19–21]. Schulze et al. have revealed that the MYC target V2 scores are associated with tumor aggressiveness and survival outcomes in ER-positive primary tumors, as well as in metastatic breast cancer [22]. Zhou et al. found that the levels of spermatogenesis-associated protein increased significantly in the serum of patients with lung cancer compared with those in healthy controls [23]. The unfolded protein response is a prosurvival mechanism triggered by accumulation of unfolded or misfolded proteins in the endoplasmic reticulum, and unfolded protein response signalling plays important roles in cancer progression [24]. Besides, the emergence of immunotherapy makes people have a new understanding of the treatment of tumor, and immune checkpoints have become a potential and effective treatment [25, 26]. In this study, the expression levels of the six immune checkpoint genes significantly increased in cluster 1 when compared to that in cluster 2. In addition, LUAD patients in cluster 1 was more sensitive to anti-PD1 therapy, suggesting that cluster 1 has the potential to determine the specific LUAD patients who are immunogenic and more responsive to immune checkpoints.

Based on the expression matrix of 131 marker genes in TCGA-LUAD, six prognosis-related genes were used to construct the prognostic model, containing CD69, CLIC6, CTSL, EPHX1, LMO3, and MS4A7. CD69 is the earliest cell surface marker of activated T cells [27]. Cibrián et al. showed that CD69 regulates the regulatory T (Treg) cell differentiation and the of IFN-*γ*, IL-17, and IL-22 secretion [28]. Martín et al. found that CD69 related to Jak3/Stat5 proteins regulates Th17 cell differentiation [29]. de la et al. have revealed that CD69 participates in immune cell homeostasis and regulates T cell-mediated immune response by controlling Th17 cell differentiation [30]. Previous studies have found that CLIC6 is overexpressed in breast cancer and endometrial cancer [31, 32]. CTL upregulation is common in a variety of human tumors and has been widely correlated to metastasis, invasiveness, and poor prognosis [33]. EPHX1 plays significant roles in the detoxification and activation of tobacco-derived carcinogens, as well as lung cancer, and the low activity genotype of EPHX1 gene is related to the reduction of lung cancer risk in whites [34]. Sun et al. have revealed that LMO3 advances human preadipocyte differentiation through increasing PPAR*γ* transcriptional activity [35]. Qiu et al. have shown that LMO3 advances gastric cancer cell proliferation and invasion via Akt-GSK3*β* and Akt-mTOR signaling [36]. Chen et al. have found that microRNA-382 inhibits cancer cell metastasis and growth in nonsmall cell lung cancer through targeting LMO3 [37]. Sun et al. uncovered that low mRNA expression of MS4A7 was correlated to better OS in all gastric cancer patients [38]. Thus, we suspected that these six cell differentiation trajectory-related genes played vital roles in the LUAD patient survival.

Besides, the prognostic model could efficiently stratify the patient outcomes and was verified in an independent dataset. Patients in the high-risk group had poorer prognosis than those in the low-risk group. The AUCs survival times at 1-year, 3-year, and 5-year were 0.706, 0.685, and 0.639 for TCGA dataset and 0.76, 0.65, and 0.616 in the GSE42127 dataset, suggesting that the performance of the gene signature was reliable. In addition, the stage and risk score were independent prognostic factors in patients with LUAD for clinical-decision support. Moreover, a nomogram was built with the stage and risk score. Because of its intuitive visual performance and personalized application, the nomogram has become a popular tool for tumor prognosis [39, 40]. Consistently, in this study, the nomogram might accurately estimate the survival probabilities for LUAD patients.

Also, this study has numerous limitations. First, the data analyzed in this study were acquired from public databases, and external validation was needed. Second, the clinical features obtained from TCGA database are limited, and potential prognostic factors, such as smoking, targeted drug therapy, and personal history, should be considered in this study. Third, the six cell differentiation-related genes and the prognostic model analyzed in this study are needed to validate in clinical samples.

## 5. Conclusion

In summary, this study has constructed a reliable prognostic risk model that is closely associated with cell differentiation trajectory, which can better predict survival and provide insights into potential markers for therapeutic strategies in lung cancer patients.

## Figures and Tables

**Table 1:** Cell type identification of each cluster.

Cluster	Annotate
0	AT2 stage IA
1	Other
2	Nonclassical monocyte
3	Mesenchymal stem cell
4	Dendritic cell progenitor cell
5	Dendritic cell
6	Endothelial
7	AT2 stage IA
8	Dendritic cell, T cell
9	Fibroblast
10	CD8 + T cell
11	Plasmacytoid dendritic cell
12	Transitional B cell
13	B cell

**Table 2:** Independent prognostic analysis.

Id	HR	HR.95L	HR.95H	*P* value
Univariate cox analysis				
Age	1.0045	0.9890	1.0202	0.5696
Sex	1.1161	0.8249	1.5099	0.4764
T	1.5187	1.2614	1.8284	1.02*E*-05
N	1.6598	1.3946	1.9755	1.17*E*-08
M	0.9041	0.7561	1.0811	0.2693
Stage	1.6150	1.3970	1.8671	9.26*E*-11
Riskscore	1.5038	1.3482	1.6774	2.48*E*-13

Multivariate cox analysis				
Id	Hr	HR.95L	HR.95H	*P* value

—	—	—	—	—
—	—	—	—	—
T	1.1939	0.9740	1.4635	0.08800
N	1.2501	0.9885	1.5810	0.06245
—	—	—	—	—
Stage	1.3407	1.0844	1.6575	0.00676
Riskscore	1.4980	1.3333	1.6829	1.02*E*-11

## Data Availability

The data that support the findings of this study are available in TCGA and GEO databases.
